# Biomonitoring Exposure and Early Diagnosis in Silicosis: A Comprehensive Review of the Current Literature

**DOI:** 10.3390/biomedicines11010100

**Published:** 2022-12-30

**Authors:** Iulia-Maria Căluțu, Raluca-Andreea Smărăndescu, Agripina Rașcu

**Affiliations:** 1 Doctoral School, Carol Davila University of Medicine and Pharmacy, 020021 Bucharest, Romania; 2 Clinical Department 5, Carol Davila University of Medicine and Pharmacy, 020021 Bucharest, Romania; 3 Department of Occupational Medicine, Colentina Clinical Hospital, 020125 Bucharest, Romania

**Keywords:** silicosis, biomarkers, early diagnosis, monitoring, occupational history

## Abstract

Silicosis is a particular form of lung fibrosis attributable to occupational exposure to crystalline silica. The occupational exposure to crystalline silica also increases the risk of chronic obstructive pulmonary disease (COPD), cancer and lung infections, especially pulmonary tuberculosis. Silicosis is currently diagnosed in previously exposed workers by standard chest X-ray, when lesions are visible and irreversible. Therefore, it would be necessary to find specific and non-invasive markers that could detect silicosis in earlier stages, before the occurrence of X-ray opacities. In this narrative review, we present several diagnostic, monitoring and predictive biomarkers with high potential in the management of silicosis, such as: pro- and anti-inflammatory cytokines (TNF (Tumour necrosis factor-α), IL-1 (Interleukin-1), IL-6, IL-10), CC16 (Clara cell 16, an indirect marker of epithelial cell destruction), KL-6 (Krebs von den Lungen 6, an indirect marker of alveolar epithelial damage), neopterin (indicator of cellular immunity) and *MUC5B* gene (*Mucin 5B*, a gel-forming mucin in mucus). Studies have shown that all the aforementioned markers have a high potential for early diagnosis or evaluation of progression in silicosis and represent promising alternatives to radiology. We consider that a multicentric study is needed to evaluate these biomarkers in correlation with occupational history, histopathological examination, imaging signs and pulmonary functions tests on large groups of subjects to better evaluate the accuracy of the presented biomarkers.

## 1. Introduction

Silicosis is a collagenous pneumoconiosis caused by long-term exposure to crystalline silica-rich dust. More precisely, silicosis is a type of pulmonary fibrosis caused by inhaled silica particles [1]. For crystalline silica particles to be biologically active, they must be small enough (“respirable”) to reach the distal airways and alveoli; [2] therefore, their diameter should be less than 5 μm [3]. In addition, the concentration of crystalline silica in inhaled particles must reach a certain threshold (usually >10%), and the exposure time must be at least 5 years [1]. The development of silicosis is a chronic and progressive process; therefore, once it occurs, it is irreversible. There are several individual characteristics and behaviours that increase the risk of occurrence, such as: pre-existing pathology of the respiratory tract (pulmonary tuberculosis, chronic rhinitis and bronchitis, etc.), genetic polymorphisms, alcohol intake, smoking and physical activity [1,4].

## 2. Occupational Exposure

Crystalline silica is the aetiological agent involved in the development of silicosis. It is a mineral found in the earth’s crust, [5] where it occurs in two distinctive forms: crystalline (quartz) and amorphous (diatom) [1]. Both crystalline and amorphous forms transform into tridymite at high temperatures (800–1000 °C), which, in turn, subjected to even higher temperatures (1100–1400 °C), transforms into cristobalite. These three forms (quartz, tridymite, cristobalite) are the main aetiological agents of silicosis, whose fibrogenic potential increases in the mentioned order. In Romania, the limit values of exposure for quartz are 1 mg/m^3^, while for tridymite and cristobalite the value is 0.5 mg/m^3^ [1,2,4]. The National Institute for Occupational Safety and Health (NIOSH) recommends the exposure limit to be less than 0.5 mg/m^3^ for any allomorphic shape [6].

According to the National Institute of Public Health, in Romania, silicosis registered a significant decrease in the number of new cases in 2019: 87 compared to 149 in 2018 and ranked second in the overall morbidity structure. Later, in 2021, a slight increase in overall number of cases was observed: 55 compared to 30 in 2020. Between 1998 and 2021, the mean value of new silica cases was 294.3 per year [7].

Epidemiological studies have shown that crystalline silica exposure is associated with increased mortality and morbidity rate [8] due to silicosis, chronic obstructive pulmonary disease (COPD) and lung cancer [9]. Worldwide, in 2019, 655.7 thousand disability-adjusted life years were attributed to silicosis [10]. In 2017, 23 695 cases of silicosis were reported globally. Between 1990 and 2017, some geographic regions reported a decrease in the number of silicosis cases (mostly in Europe), but certain regions such as North and South Africa, China and sub-Saharan Africa reported an upward trend [11].

NIOSH identifies the following activities as occupations at risk: (1) manufacturing of glass, pottery, ceramics, bricks, concrete and artificial stone, (2) abrasive blasting, (3) foundry work, (4) hydraulic fracturing, (5) stonecutting and stone countertop, (6) rock drilling, (7) quarry work, (8) tunneling, (9) construction, (10) mining, (11) oil and gas extraction and (12) dentistry [6].

Given the evolution of society and technological processes, physicians must pay particular attention in diagnosing silicosis both in those previously exposed in the former industries, even if some of them have disappeared in certain countries (e.g., mining, foundries) and in those placed in newly emerging professions (e.g., sandblasting jeans, artificial stone benchtop) [9,12].

## 3. Materials and Methods

**Figure 1:**
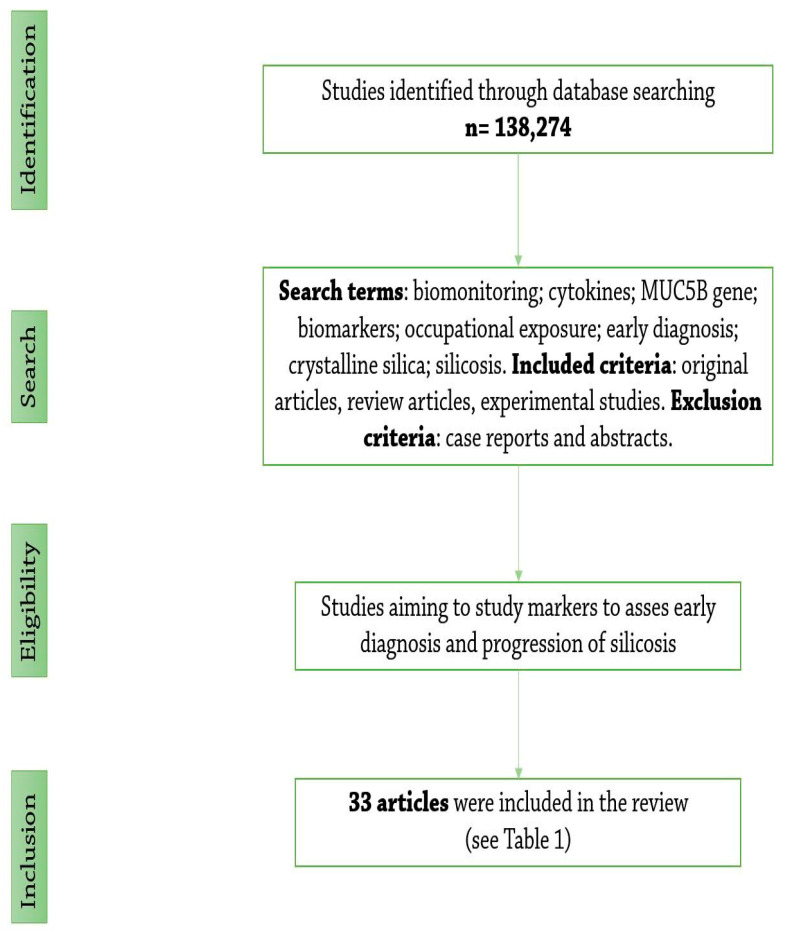
Flowchart of the literature search.

This article is a narrative review designed to evaluate and present several diagnostic, monitoring and predictive biomarkers used for patients with occupational exposure to crystalline silica. For this review, existing literature was selected from various databases such as Pubmed, Scopus, ScienceDirect and Google Scholar. After a thorough analysis as shown in Figure 1 (*n* = 138 274 articles), we selected 33 studies presented in Table 1, aiming to study markers to assess early diagnosis and progression of silicosis. The cytokines (TNF-α (tumour necrosis factor-α), IL-1 (Interleukin-1), IL-10, IL-6), CC16 (Clara cell 16), KL-6 (Krebs von den Lungen 6), *MUC5B (Mucin 5B)* gene and neopterin were selected as points of interest for further search. We used the names of the aforementioned markers followed by the terms “silicosis”, “inflammation”, “anti-inflammatory”, “immune dysregulation”, “physiopathology”, “evolution”, “early diagnosis”, “genetic polymorphisms”, and “treatment” in different permutations. For each item, we have summarised the biological mechanisms using the search engines described above and the national literature. We then presented a selection of clinical and experimental studies that evaluated several biomarkers for their potential value in the early diagnosis and evolution of silicosis. We did not limit the time range, although more recent studies were preferred.

## 4. Health Effects

Silicosis is the most common pneumoconiosis and is classified based on the radiological findings into two categories: simple (<10 mm diameter opacities) and complicated (>10 mm diameter opacities). The pathogenesis of silicosis is based on three theories: macrophage destruction, inflammation leading to fibrosis and immunological mechanisms [4].

**Figure 2:**
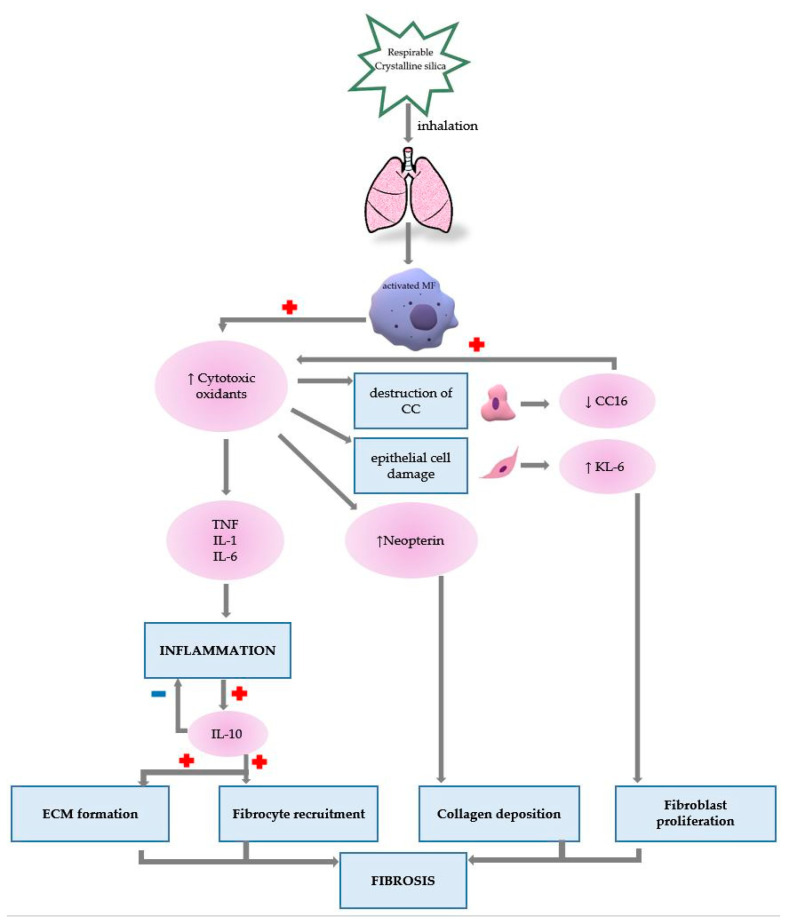
The role of presented biomarkers in silicosis pathogenesis (MF—macrophage, TNF—Tumour necrosis factor, IL—Interleukin, CC—Clara cell, KL-6—Krebs von den Lungen 6).

Macrophage destruction provides the foundation for the onset of inflammation, fibrosis and immunological processes. Crystalline silica particles are phagocytosed by macrophages leading to a series of events that present, in chronological order as follows: (1) rupture of the phagosome wall under the action of lysosomes, (2) release of the contents into the macrophage cytoplasm, (3) disintegration of the macrophage, (4) release of particles and enzymes into the extracellular fluid, (5) resumption of the process. Once activated by respirable-size silica, the macrophages will initiate the inflammatory process, collagen hypersynthesis and immunological processes (Figure 2) [1,4].

Given that the histopathological onset of the disease has no radiological signs (there is a delay between histopathological onset and radiologically visible lesions) [1,3], potential biomarkers useful for early diagnosis have been suggested. We have summarised the most relevant aforementioned biomarkers, as highlighted by specialty literature hitherto.

## 5. Biomarkers

### 5.1. TNF

Alveolar macrophages, the first defence line against foreign substances, ingest inhaled silica, leading to cell death and extracellular silica release. The silica is then captured by other macrophages, leading to a repetitive cycle that maintains the inflammatory process. Macrophages release a number of mediators such as cytotoxic oxidants, arachidonic acid metabolites and inflammatory cytokines including TNF-α, IL-1. These mediators initiate the influx of inflammatory cells and induce their infiltration into the alveolar wall, releasing proteolytic enzymes and toxic oxygen derivatives, leading to cell damage and destruction of the extracellular matrix [45].

TNF is a glycoprotein produced primarily by activated macrophages, but also by other cells: mast cells, lymphocytes, fibroblasts, granulocytes, NK (natural killer) cells [46]. The effects of TNF include activation of neutrophils, macrophages, B and T lymphocytes and stimulation of immunoglobulin synthesis. It also induces tumour cell death by necrosis or apoptosis and stimulates synthesis of other cytokines: IL-1, IL-6, and IFN (Interferon). TNF-α stimulates the synthesis and deposition of extracellular matrix and collagen synthesis, hence promoting the development of fibrosis [13]. Therefore, TNF-α plays an important role in maintaining the inflammatory process by activating macrophages, stimulating the production of inflammatory proteins (cytokines) and stimulating T lymphocytes [4]. TNF-α holds several important roles in silicosis, including triggering the influx of inflammatory cells and release of other cytokines [13].

Studies showed that TNF-α levels are elevated before the onset of clinical signs associated with silicosis, making TNF-α a valuable option for early diagnosis [14,47].

Data provided from one study that included 30 controls (healthy individuals), 28 silica-exposed individuals (without clinical disease), and 30 silica subjects showed that TNF-α plasma levels were elevated in exposed workers (*p* < 0.05) and significantly higher in silicosis patients than in the healthy subjects group (*p* < 0.01), proving that TNF-α is a contributor in the pathogenesis of silicosis [13].

Evidence from TNF-deficient mice, which are resistant to the development of silica-induced fibrosis, supports the idea that TNF-α plays a significant role in the development of pulmonary fibrosis [16]. Local release of IL-1 and TNF-α on cultures of monocytes and macrophages in humans has been shown to be consistent with disease pathogenesis [39].

Using specific TNF inhibitors that alter the NF-collagen (non-fibrillar), F-collagen (fibrillar), and P4H (Prolyl 4-hydroxylase) gene response (fibrogenic gene expression levels), it was shown that in quartz-treated explant cultures, compared to controls, there was an overexpression of TNF and silica-induced sponge collagen production [36]. Anti-TNF may reduce silica-induced pulmonary inflammation by lowering NF-κB (nuclear factor kappa B), signalling, oxidative stress, and TNF-α, suggesting that anti-TNF may be used to treat silica-induced lung injury [37].

Past studies place promoter polymorphisms of TNF-α in inflammatory and infection-prone conditions [4]. Currently, there is no documented link between TNF polymorphism-related susceptibility to infections and silicosis, [23,48] but it remains open whether a direct association is possible, given the susceptibility of patients with silicosis to pulmonary tuberculosis [4]. A meta-analysis assigns that TNF -308 (11 studies) and -238 (8 studies) polymorphisms are associated with susceptibility of silicosis [17]. Our findings show that also other TNF polymorphisms are linked to silicosis. Corbett et al. identified a strong link between TNF-α polymorphisms -238 and -376 and severe silicosis [18].

A study conducted on subjects working in a cement factory found that individuals who had genetic variation at the TNF -308 gene loci were more susceptible to develop silicosis. These findings were sustained by the higher amount of TNF-α produced by TNF -308 gene variation subjects (*p* = 0.004) [19].

Overall, the studies showed that increased TNF-α levels in silica-exposed workers without clinical signs of the disease could be a potential biomarker for diagnosing early silicosis. There are still two issues that need further assessment in the interpretation of these results. First, genotyping of the TNF polymorphisms should be taken into account in all studies referring to TNF as a biomarker for silicosis, as TNF was shown to determinate the susceptibility to silicosis, and of developing a complicated form (progressive massive fibrosis).

Second, the updated classification of TNF/TNF-α should be used. TNF was originally attributed to two molecules, TNF-α, a monocyte-derived tumour necrosis factor, and TNF-β, a lymphocyte-derived tumour necrosis factor [46]. Then, at the Seventh International TNF Congress (17–21 May 1998; Hyannis, Massachusetts), the name “TNF-β” was changed to “lymphotoxin-α”. Concomitantly, “TNF-α” became an unnecessary term, with the same meaning as the original term, “TNF”, which was re-established as the official cytokine name [49]. Even though it was renamed more than 20 years ago, TNF continues to be used as TNF-α in a large number of current scientific studies, leading to some sort of misunderstanding of which molecule authors are referring to.

In one experimental study, anti-TNF treatment showed promising potential in reducing silica-related inflammation. However, clinical studies are missing, and at this point we cannot conclude on the efficacy in silicosis.

### 5.2. IL-1

Macrophages activated by respirable-size crystalline silica produce mediators and initiate the inflammatory process. IL-1 produces synergistic effects with other pro-inflammatory cytokines, such as TNF-α and IL-6. Cytokines secreted by activated macrophages (IL-1, IL-6, IL-12, IL-18) will in turn attract and activate T lymphocytes, leading to stimulation of B lymphocytes by the latter (via IL-11 and IL-14) and initiation of the immunological response. The persistence of silica particles in the lung tissue induces a chronic activation of all these cells, ensuring an ongoing inflammatory process [4].

Experimental animal studies and clinical trials show that TNF-α and IL-1 are important in the regulation of fibrotic mediators in silicosis. Inter-individual differences in IL-1 and TNF-α production sustain the idea that silicosis and the progression to its complicated form are linked to the host’s genetic predisposition to produce these proteins, since in inflammatory diseases, some allelic variants were found to be overexpressed. For example, IL-1 gene polymorphism (IL-1RA +2018) exhibits independent and correlated effects with the susceptibility and severity of silicosis in exposed individuals, thus the occurrence of silicosis would not only rely on the intensity, duration and time of exposure, but also on the cytokine polymorphism [24,39].

These results are in accordance with the findings of Yucesoy B et al., where IL-1RA +2018 was significantly increased in patients with moderate and severe silicosis, suggesting that this variant mainly impacts disease susceptibility [39].

A study conducted on a group of 99 subjects exposed to crystalline silica in a Turkish ceramic plant showed significantly increased levels of the studied interleukins in serum, including IL-1, in comparison to the control group of 81 subjects. Moreover, older subjects had more elevated serum IL-1α values compared to younger subjects [20].

Studies evaluating the relationship between IL-1β levels in blood and exposure to crystalline silica showed significant correlations between the profusion of opacities in the silica group compared to healthy subjects (*p* < 0.05) [21,38].

Although the homology between IL-1α and IL-1β is 27%, [50] they bind to the same receptor, IL-1R1 (IL-1 type 1 receptor), and induce the same biological functions [50,51]. Hence, in silicosis, IL-1 is involved in collagen deposition and modulation of PDGF (platelet-derived growth factor) activity [40]. Furthermore, a study conducted on cultures of cells sampled from rodent lung tissue showed that after exposure to crystalline silica, IL-1α is rapidly released by alveolar macrophages, stimulating the production of IL-1β, thus promoting lung inflammation [41].

Alongside TNF, results showed that IL-1 polymorphisms genotyping, especially IL-1RA +2018, can be related to the susceptibility and severity of silicosis. The correlation between the levels of IL-1β and the density of radiological opacities in silica patients were reported in only one study and needs to be confirmed in other cohorts. Therefore, further studies on larger groups of subjects are needed in order to eventually implement IL-1β in monitoring the disease and IL-1 in the screening protocol of exposed individuals and determine the specific IL-1 polymorphisms linked to developing silicosis.

### 5.3. IL-10

Anti-inflammatory cytokines are a group of immunoregulatory molecules adjusting the pro-inflammatory cytokine response. To control the immune response, cytokines act together with certain specific cytokine inhibitors and soluble cytokine receptors. One of the most important anti-inflammatory cytokines is IL-10. IL-10 is an important controller of the differentiation and proliferation of various immune cells and moderating and even suppressing inflammatory reactions [52].

In silicosis, IL-10 is elevated but has a dual effect: on one side, IL-10 limits the amplitude of the inflammatory response by suppression of the production IL-1β, IL-6 and TNF-α in monocytes and macrophages [53]. On the other side, by inducing the fibrotic process, IL-10 contributes to the extension of the pneumoconiotic lesions.

Kurniawidjaja LM started from the hypothesis that the inflammatory process stimulates the production of IL-10, which has an anti-inflammatory role. The TNF-α over IL-10 ratio was evaluated, and the results showed a ratio less than 1 had a protective effect for developing silicosis. The most probable explanation was that the anti-inflammatory effect of IL-10 outweighs the inflammatory effect of TNF-α. If TNF-α/IL-10 ratio is supraunitary, IL-10 is not able to suppress the pro-inflammatory effect of TNF-α, suggesting that the risk factor for silicosis should be derived from this ratio and not from the independent values of TNF-α and IL-10. The significant difference between the TNF-α/IL-10 ratio values was independent of the TNF-α genetic variation [49].

### 5.4. IL-6

IL-6 is a multifunctional cytokine and plays an essential role in inflammation and immunity. In pulmonary diseases, elevated levels of IL-6 are found in bronchoalveolar lavage fluid, lung tissue and blood. IL-6 facilitates lung infiltration with inflammatory cells by inducing cell expression of adhesion molecules on inflammatory cells and plays a role in regulating fibrosis by modulating the expression of Th2 cytokines [54].

IL-6 controls the production of IL-1 and TNF-α and is known as a primary mediator of the acute phase response and also has anti-inflammatory effects. In the presence of TGF-β (transforming growth factor-β), IL-6 inhibits the development of regulatory T cells and promotes Th17 differentiation, which produces IL-17 [16].

Braz NFT et al. and Blanco-Pérez JJ et al. investigated various cytokines; one of the major findings of both studies was that higher serum levels of IL-6 were found in silicosis patients and in those exposed to crystalline silica than in non-exposed healthy individuals [15,16].

In a recent clinical study, a group of silicosis patients was divided into two categories to evaluate a potential treatment for silicosis (acetylcysteine + tetrandrine) and its effect on serum IL-6 and TNF-α levels. Tetrandrine together with N-acetylcysteine was used on a routine basis to treat patients in the observation group, while the control group received standard, symptomatic treatment. Before therapy, no noticeable difference between the blood levels of IL-6 and TNF-α (*p* > 0.05) of the two groups was observed. After treatment, the levels of the aforementioned cytokines were reduced in both groups, but in the observation group the reduction was considerably lower (*p* < 0.05). Tetrandrine together with acetylcysteine may contribute well together to enhance the clinical therapeutic impact in silicosis and reduce the severity of inflammation. The clinical therapeutic effect was assessed by determining FVC (forced vital capacity), FEV1 (forced expiratory volume in 1 s) and RR (respiratory rate). FVC, FEV1 and RR showed an improvement after treatment, but no correlation was performed with the chest X-ray or computed tomography. Based on these results, the authors concluded that peripheral blood IL-6 and TNF-α levels are important for silicosis management, and their detection could reduce the number of X-rays as a follow-up procedure [22].

Along with TNF-α and IL-1, IL-6 has long been considered a pro-inflammatory cytokine produced by lipopolysaccharide. IL-6 is frequently used as a sign of systemic pro-inflammatory cytokine activity. IL-6 possesses pro-inflammatory and anti-inflammatory characteristics, as do many other cytokines, with the acute phase protein response being strongly induced by IL-6. IL-6 has a reduced impact on the production of anti-inflammatory cytokines, such as IL-10 and TGF- β, and reduces the secretion of pro-inflammatory cytokines. In addition to enhancing IL-1Ra (IL-1 receptor antagonist) production and soluble TNF receptor release, IL-6 increases glucocorticoid synthesis. IL-6 also prevents the synthesis of pro-inflammatory cytokines such as GM-CSF (granulocyte macrophage colony-stimulating factor), IFN-γ and MIP-2 (macrophage inflammatory protein-2) [55].

IL-6 showed promising results in diagnosing silicosis, including its incipient stage, when opacities are not radiologically visible. However, IL-6 is a cytokine secreted as a response in many other inflammatory reactions (infections, exposure to other particles, etc.), and these circumstances have to be excluded in the individual judgement of this biomarker significance.

In already diagnosed silicotic patients, the dynamics of the IL-6 could reduce the number of chest X-rays and could be used in monitoring the management of the disease.

### 5.5. CC16

Clara cell protein (CC16) is a protein secreted by Clara cells, whose name comes from its molecular weight of 16 kD. It is mainly found in the distal respiratory tract, more specifically in the terminal bronchioles [25,42,56]. This protein has an anti-inflammatory, antioxidant, anti-fibrotic and immunosuppressive role [25,57]. Airways inflammation could lead to a reduction in the number of Clara cells, and the degree of reduction may reflect the epithelial cell damage over time [58]. Several studies have even suggested CC16 as a peripheral biomarker of lung epithelial destruction [25,26,27,28]. Different levels of Clara cell damage could lead to a decrease in function, especially in their anti-inflammatory capacity. A potential reason could be the silica dust’s ability to cause inflammatory damage to the lungs; as this inflammation gradually increases with prolonged exposure, it results in decreased secretion of Clara cells via cellular damage. Toxins released by activated phagocytes and free radicals will also contribute to this destruction [26].

One study showed a decrease in CC16 levels in BALF (Broncho-alveolar lavage fluid) in the silicosis group with small opacities (<10 mm) compared to the control group [3,26]. Moreover, the authors reported lower CC16 levels in patients with simple silicosis compared to the group with complicated silicosis (progressive massive fibrosis) (*p* < 0.05) [3,26]. This result was attributed by the authors to a possible self-repair process of epithelial cells [26] but, to the best of our knowledge, without experimental evidence, such as a lung biopsy, to support the assumption.

Another study comparing three groups (silicosis, exposed and control group) showed that the serum levels of CC16 were lower in the silicosis group, followed by the exposed group, and the highest levels were in the control group (*p* < 0.001) [25].

A 2020 study suggests that a CC16 serum value below 7.0 ng/mL in workers with an occupational history of crystalline silica exposure could represent a potential marker for detection of silicosis in early stage [27].

Sarkar K et al. investigated the CC16 in the serum of 117 silicosis subjects and 32 non-exposed individuals. The results of the study showed an inversely proportional relationship between the degree of lung damage on chest X-rays and CC16 serum values. The study also suggests that a cut-off value of 9 ng/mL can be correlated with early silicosis [28].

Although the cut-off values of the two studies differ, it is a promising start in recruiting peripheral biomarkers for the diagnosis of early-stage silicosis. Considering the limitations discussed, more studies are needed to accurately determine the cut-off value of CC16, preferably on larger groups of subjects with different radiological stages.

A study conducted on 106 subjects (68 silica-exposed and 38 healthy individuals) measured serum CC16 levels by two methods: ELISA (enzyme-linked immunosorbent assay), the standard reference method, and semi-quantitative lateral flow assay (immunochromatography). By ELISA, all subjects radiologically confirmed with silicosis had CC16 levels below 9 ng/mL, while healthy subjects showed CC16 > 9 ng/mL. In the semi-quantitative lateral flow assay, CC16 values were represented by ranges (<6 ng/mL, 6.1–9 ng/mL, >9 ng/mL), and the results by this method showed a sensitivity of 100% and specificity of 95%, compared to the ELISA results [29]. These findings propose a new approach in CC16 detection, being a much more affordable and reproducible method, which can be easily implemented as a screening method in all silica-related occupational exposure, even in less-advanced geographical areas.

All findings showed that serum and BALF CC16 levels in silica patients were significantly lower than the non-exposed groups. Moreover, serum CC16 levels were reported to decrease in accordance with silicosis stage and correlated to the FEV1/VC ratio. CC16 detection by semi-quantitative lateral flow assay should be applied to larger groups of subjects to better evaluate the sensitivity and specificity compared to ELISA in silica-exposed individuals.

### 5.6. KL-6

KL-6, also known as MUC-1 (Mucin 1), is a mucin-like glycoprotein with a high molecular weight and is shown to be expressed on type 2 pneumocytes (mostly in the cytoplasm and membrane) [59,60], Clara cells and bronchial glands [61]. Nowadays, it is known that increased levels of KL-6 in serum reflect the presence of an active alveolar epithelial damage [62]. KL-6 can promote the migration and proliferation of fibroblasts and inhibit programmed cell death (apoptosis). Thus, KL-6 could lead to pulmonary fibrosis [63].

KL-6 released by the proliferation of type 2 pneumocytes in pulmonary fibrosis related to occupational exposure to dust or fibres, as pneumoconioses, leads to an increased KL-6 serum concentration. Thus, it may stimulate fibrotic processes in patients with interstitial lung disease and raises the possibility of needing an anti-KL-6 antibody treatment [64].

A study in mice showed that after 45 days of crystalline silica exposure, the pulmonary fibrosis became observable and the level of KL-6 in serum was positively correlated with the severity of fibrotic lesions [43]. Data on human subjects showed that serum KL-6 concentrations are higher in pneumoconioses than healthy controls or exposed individuals [30].

Thus, the results specifically referring to silica are scarce. KL-6 is a potential biomarker for occupational induced fibrosis and for lung fibrosis, in general. The role in diagnosing silicosis is not yet defined.

### 5.7. MUC5B Gene

*MUC5B* gene encodes the MUC5B protein, the main gel-forming mucin in humans and mice mucus. Thus, MUC5B contributes to lubrication and viscoelasticity of lungs, saliva and cervical mucus [65].

Studies showed the *MUC5B* overexpression in the distal airways disturbs the balance required to support efficient mucociliary transport, thereby affecting mucus function. The implication of MUC5B in the development of pulmonary fibrosis proposes two hypotheses. First, the exposure to respirable dust and microparticles, and subsequently their retention in the lungs, could lead to mucociliary dysfunction. Secondly, the inflammation induced by retained substances could represent the beginning of collagen deposition through fibrotic microlesions. Another theory for the occurrence of pulmonary fibrosis linked to *MUC5B* overexpression is sustained by a decrease in the lung clearance and an increased mucus viscosity [66].

A study in mice showed that silica particles could lead not only to an alteration in the expression of *MUC5B*, but also to cilia dysfunction and excessive mucus secretion. More studies are needed to better understand if these findings are directly linked to silicosis and should also include data about *MUC5B* polymorphisms and their implication in the susceptibility of silica-related fibrosis [44].

A study conducted on a Chinese population shows that *MUC5B* rs2672794 gene polymorphism is in direct association with coal miner’s pneumoconiosis, thus *MUC5B* rs2672794 CC genotype could increase the risk of developing pneumoconiosis [31].

As *MUC5B* is a gene extensively studied for its role in lung fibrosis, in future studies, the modification of the gene expression in humans by silica exposure should be considered.

### 5.8. Neopterin

Neopterin, a pyrazinopyrimidine molecule that belongs to the pteridine class, soluble in plasma or serum, is a crucial and early indicator of cellular immunity. Dendritic cells, macrophages and monocytes that have been stimulated by IFN-γ produce neopterin. Neopterin is a useful prognostic biomarker for immunological stimulation, persistent infection, cell-mediated immunity and oxidative stress [67]. The neopterin secretion induced by IFN-γ is linked to the production of cytotoxic oxidants, making neopterin a candidate for monitoring oxidative stress, not only cellular immunity [32].

Serum neopterin levels could be used as an indicator of the silica-exposure-related impact and other occupational disorders. Elevated levels of neopterin in the serum of silicosis patients raise the possibility of its implication in cellular immunity and ongoing macrophage activation in disease pathogenesis [34]. Neopterin could be considered a potential biomarker for identifying the earliest health effects of crystalline silica [32]. However, in order to be implemented in clinical practice, further studies are needed to investigate oxidative stress parameters besides neopterin [32].

In a study of workers exposed to crystalline silica, significantly higher levels were found in exposed subjects compared to healthy individuals (*p* < 0.05). The results also demonstrated that the increased neopterin values in exposed subjects are primarily influenced by the presence of crystalline silica in the respirable fraction and are not impacted by individual characteristics or exposure time [35]. Nonetheless, the study’s analysis starts from an incomplete description of the method, without taking into account the subjects’ average exposure to crystalline silica. The study provides limited information on exposure and particularly on long-term exposure. The data rely only on the respirable fraction measured at a given time point, although subjects’ exposure in some cases was over 20 years.

Another study obtained significant statistically differences in serum and urinary neopterin levels between the exposed subjects and healthy individuals. Neopterin levels in urine and serum have been noticeably elevated in silica-exposed workers. Increased neopterin levels in blood, urine and other body fluids could indicate the level of cellular immune activation and estimate the amount of oxidative stress [33].

Overall studies showed that neopterin has a great potential as a biomarker for early detection of silicosis, but, for better accuracy, the oxidative stress parameters should also be measured.

## 6. Conclusions

Silicosis is still one of the major industrial health issues all over the world. Given the current context where the diagnosis of silicosis is established only on the basis of late and irreversible radiological changes, the lack of specific biomarkers in the screening protocol of silica-exposed patients becomes increasingly necessary.

To integrate the presented results into clinical practice and the diagnostic protocol for early silicosis, further studies are needed to investigate the cytokine profile and functional polymorphisms in silicosis patients. These results should be correlated with occupational history (exposure time, retention time, duration and intensity of exposure), histopathological exam, imaging findings and pulmonary function test results. Moreover, these results should be interpreted in a clinical context and should exclude silica-related respiratory diseases such as industrial bronchitis and possible exacerbations.

Although all the findings show tremendous potential for early diagnosis of silicosis, CC16 detection by immunochromatography seems the most promising and should be applied to larger groups of subjects to demonstrate on a much wider scale the sensitivity and specificity of the method for future introduction into clinical practice and screening protocols.

## Figures and Tables

**Table 1:** Extraction table of the 33 articles included in the narrative review (TNF—Tumour necrosis factor, OR- Odds ratio, CI—Confidence interval, IL—Interleukin, CC16—Clara cell 16, BALF—Broncho-alveolar lavage fluid, FEV1—Forced expiratory volume in 1 s, VC—Vital capacity, ELISA—Enzyme-linked immunosorbent assay, KL-6—Krebs von den Lungen 6, SP-D—Serum surfactant protein D, MMP—Matrix metalloproteinase, *MUC5B*—*Mucin 5B*, CWP — Coal workers’ pneumoconiosis, NF-κB—Nuclear factor kappa B, iNOS—Inducible nitric oxide synthase, IHC—Immunohistochemistry, TEM—Transmission electron microscopy).

Human Studies	
Biomarker	Results
TNF-α	● TNF-α is increased prior to the onset of clinically diagnosed silicosis [13].
	● TNF-α serum levels showed no differences between control groups and silica-exposed patients [14,15].
	● TNF-α polymorphisms showed independent and interrelated effects on susceptibility and severity of silicosis in underground miners [16].
	● TNF −308A/G and −238A/G polymorphisms are associated with susceptibility to silicosis (an overall OR = 2.09 (1.23–3.55)), especially in Asians (OR = 3.04, CI = 1.23–7.51) [17].
	● No significant differences between miners with less severe silicosis and controls at any loci in the TNF-alpha promoter region, but miners with severe silicosis were significantly more likely than controls to have -238A and -376A. Additionally, the -308A allele was significantly more prevalent in subjects with severe silicosis [18].
	● Genetic variation on TNF- α on locus -308 in Indonesia was significantly (*p* = 0.02) higher on silicosis (13.45%) than non-silicosis (5.45%), but lower than silicosis in Africa and US miners. A risk factor for silicosis was a TNF- α:IL-10 ratio higher than 1, which resulted in a more rapid deterioration in pulmonary function than a ratio less than 1 [19].
	● Workers had significantly higher serum levels of TNF-α compared to controls [20,21].
	● There was significant correlation between the density of radiological findings with small opacity and serum levels of TNF-α (rho = 0.306, *p* < 0.01) [21].
	● TNF-α and IL-6 levels decreased following treatment with acetylcysteine and tetrandrine tablets (*p* < 0.05), with values in the observation group being considerably lower than those in the control group (*p* < 0.05) [22].
IL-1	● IL-1β levels showed no differences between unexposed healthy subjects and silica-exposed patients [14].
	● IL-1 polymorphisms showed independent and interrelated effects on susceptibility and severity of silicosis in underground miners [16].
	● On patients with a history of exposure to silica and with a previous recorded history of tuberculosis (Tb), there showed a strong association between the IL1A -889C allele or the CC genotype and susceptibility to Tb [23].
	● This meta-analysis found a significant association between IL-1RA +2018 polymorphism and increased silicosis risk [24].
	● Workers exposed to silica had significantly higher serum levels of IL-1α and IL-1β compared to non-exposed [20].
	● There was significant correlation between the profusion of radiological findings with small opacity and serum levels of IL-1β (rho = 0.218, *p* < 0.05) [21].
	● IL-1β plasma levels showed no differences between control groups and silica-exposed patients [15].
IL-10	● Workers exposed to silica had significantly higher serum levels of IL-10 compared to non-exposed [20].
	● There was a positive correlation between IL-10 and cardiorespiratory capacity and lung function; however, there was a negative correlation with the quality-of-life total score. IL-10 plasma levels showed no differences between control groups and silica-exposed patients [15].
IL-6	● In subjects exposed to silica, with and without silicosis, the levels of serum IL-6 were higher than in unexposed healthy subjects [14,20,21].
	● There was no significant difference in serum IL-6 levels between patients with silicosis and silica-exposed group [21].
CC16	● Levels of CC16 in silica group were lower than in exposed group. In non-exposed group (controls), the levels of CC16 were higher than silica and exposed group (*p* < 0.001) [25].
	● CC16 levels in BALF were lower in simple silicosis compared to the non-exposed group. The highest values of CC16 in BALF were observed in complicated silicosis group, compared to simple silicosis group and controls (non-exposed) (*p* < 0.05). The FEV1/VC ratio was positively correlated with CC16 in all groups [26].
	● CC16 serum levels were lower in complicated silicosis than silica-exposed group, followed by the control group (non-exposed) (*p* < 0.01) [27].
	● Serum CC16 values were reported to decrease in accordance with silicosis stage [28].
	● The serum of 68 silicosis subjects and 38 non-exposed subjects were tested to determine the CC16 values by 2 methods: ELISA and semi-quantitative lateral flow assay. By ELISA, all the X-ray confirmed silicotic subjects had CC16 levels < 9 ng/mL, while the non-exposed group had CC16 concentration > 9 ng/mL. Compared to ELISA, the sensitivity of the semi-quantitative lateral flow assay was 100% while the specificity was 95% [29].
KL-6	● The combination of KL-6, SP-D, and MMP-2 showed a sensitivity of 83% and a specificity of 62% in diagnosing both asbestosis and silicosis. These biomarkers were highest in asbestosis group, followed by silicosis group [30].
*MUC5B* gene	● The *MUC5B* rs2672794 CC genotype was associated with a significantly increased risk of CWP, compared with the TT genotype [31].
Neopterin	● Serum and urinary neopterin levels were higher in silica-exposed group than the non-exposed group (*p* < 0.001) [32,33]. Silica-exposure concentration was positively correlated with urinary and serum neopterin levels [32].
	● In silicosis group, serum neopterin levels were higher than non-exposed group (*p* < 0.05); no significant differences were observed between simple and complicated silicosis [34].
	● In silica-exposed group, the levels of serum neopterin were higher than the non-exposed group (*p* < 0.05) [35].
**Experimental studies**	
**Biomarker**	**Results**
TNF-α	● TNF pro-inflammatory cytokine overexpression in silica-induced sponge collagen biosynthesis was shown in quartz-treated explants as compared with controls using specific TNF inhibitors (pentoxifylline -PTX, which inhibits TNF production and SPD304, which inhibits TNF signalling) and affecting the fibrogenic gene response [36].
	● Silica installation increased the lung tissues inflammation reaction, oxidative stress and severity of pulmonary fibrosis. Infliximab administration notably improved silica-induced lung pathological alterations (collagen deposition, inflammatory cells), decreased the TNF-α inhibited NF-κB signalling, as well as the oxidant status (iNOS) [37].
	● Cells treated with silica dust samples from metal mines showed significantly higher levels of TNF-α expression compared to untreated cells. In comparison to samples from metal mines, silica dust samples from pottery factory and quartz showed reduced levels of TNF-α expression at all concentrations [38].
IL-1	● The thymocyte proliferation factor produced by silica-treated monocytes, which has all the biochemical properties of IL-1, is capable of stimulating fibroblast proliferation. Silica, a substance which leads to macrophage-mediated fibrosis in vivo, stimulates the release of IL-I; IL-l has identical properties to the factor that modulates the proliferation of fibroblasts [39].
	● The level of IL-1β in cell cultures was found to be consistent with the incidence of silicosis among dust-exposed workers [38].
	● In IL-1β ^−/−^ mice and iNOS ^−/−^ mice, IL-1β and NOS activity are crucial to the process of silica-induced apoptosis and lung inflammation [40].
	● In silica-treated mice, the lung production of pro-IL-1β and neutrophilic inflammation occurred after the early release of IL-1α from the alveolar area. Neutralization or deletion of IL-1α decreased neutrophil recruitment and IL-1β production in mice exposed to silica [41].
IL-6	● IL-6 levels were increased by quartz and silica dust samples; however, there were no significant differences in comparison to the untreated control [38].
CC16	● Decrease in CC16 expression in relation to silica exposure time (*p* < 0.01) in BALF of mice. In silica group, levels of CC16 were significantly lower than control group (non-exposed mice) [42].
KL-6	● In silica-exposed mice, the pulmonary fibrosis started on the 45th day. The levels of KL-6 in BALF were correlated with the stage of silicosis [43].
*MUC5B* gene	● In silica-exposed mice, using histological analysis, IHC, TEM, mucociliary alteration of the airway epithelium was observed. Silica dust increased the MUC5B production in submucosal glands between day 1 and day 84, while on the surface of airway epithelium the *MUC5B* expression decreased from day 28 to 84 (*p* < 0.01) [44].

## Data Availability

Not applicable.
